# Complement receptor 3 mediates renal protection in experimental C3 glomerulopathy

**DOI:** 10.1016/j.kint.2015.11.024

**Published:** 2016-04

**Authors:** Thomas D. Barbour, Guang Sheng Ling, Marieta M. Ruseva, Liliane Fossati-Jimack, H. Terence Cook, Marina Botto, Matthew C. Pickering

**Affiliations:** 1 Centre for Complement and Inflammation Research, Imperial College, London, UK; 2 Centre for Experimental Medicine and Rheumatology, Queen Mary University of London, London, UK

**Keywords:** complement, glomerulonephritis, macrophages

## Abstract

C3 glomerulopathy is a complement-mediated renal disease that is frequently associated with abnormalities in regulation of the complement alternative pathway. Mice with deficiency of factor H (*Cfh*^*–/–*^), a negative alternative pathway regulator, are an established experimental model of C3 glomerulopathy in which complement C3 fragments including iC3b accumulate along the glomerular basement membrane. Here we show that deficiency of complement receptor 3 (CR3), the main receptor for iC3b, enhances the severity of spontaneous renal disease in *Cfh*^*–/–*^ mice. This effect was found to be dependent on CR3 expression on bone marrow–derived cells. CR3 also mediated renal protection outside the setting of factor H deficiency, as shown by the development of enhanced renal injury in CR3-deficient mice during accelerated nephrotoxic nephritis. The iC3b–CR3 interaction downregulated the proinflammatory cytokine response of both murine and human macrophages to lipopolysaccharide stimulation *in vitro*, suggesting that the protective effect of CR3 on glomerular injury was mediated via modulation of macrophage-derived proinflammatory cytokines. Thus, CR3 has a protective role in glomerulonephritis and suggests that pharmacologic potentiation of the macrophage CR3 interaction with iC3b could be therapeutically beneficial.

C3 glomerulopathy (C3G) is a category of kidney diseases that includes dense-deposit disease, C3 glomerulonephritis, and complement factor H–related protein 5 (CFHR5) nephropathy.^1^ C3G is defined by the presence of isolated or dominant complement C3 within the glomerulus. It is frequently due to uncontrolled C3 activation via the complement alternative pathway, which may be genetic or acquired. Causes include deficiency or dysfunction of factor H (FH), the major plasma alternative pathway regulatory protein. Progression to end-stage kidney disease occurs in ∼40% of patients after 10 years, and recurrence after renal transplantation is common. At present, there is no proven treatment for C3G, although the C5 inhibitor eculizumab may have a role based on a small prospective trial and several case reports.^2^

Mice with homozygous deficiency of FH (*Cfh*^*–/–*^) have proved to be an informative experimental model of C3G.^3^ *Cfh*^*–/–*^ mice spontaneously develop low plasma C3 and C5 levels and linear staining of C3 and C9 along the glomerular basement membrane (GBM). The development of glomerular C3 deposition is absolutely dependent on activation of C3 through the alternative pathway.^3^ Complement C3 along the GBM in these animals includes iC3b, a cleavage product of C3b.^4^, ^5^ Complement receptor 3 (CR3), also known as Mac-1 (CD11b/CD18, α_M_β_2_), is the main leukocyte receptor for iC3b.^6^, ^7^ CR3 is a β_2_–integrin receptor expressed mainly on neutrophils, monocytes and macrophages, and dendritic cells.^8^ The α-chain (CD11b), encoded by the *ITGAM* gene, confers binding of CR3 to a wide range of endogenous and pathogen-derived ligands. CR3 mediates diverse cellular functions including phagocytosis of iC3b-coated particles, cytotoxicity, chemotaxis, and cell adhesion,^9^ which play a critical role in regulating inflammation and antimicrobial immunity. *In vitro* and *in vivo* observations have also revealed a role for CR3 in toll-like receptor (TLR)–triggered innate immune responses, but the nature of this cross-talk remains controversial, with some studies demonstrating that CR3 can promote TLR-induced inflammation,^10^, ^11^, ^12^ whereas others have reported negative roles for this integrin in TLR responses.^13^, ^14^

CR3 has been implicated in the pathophysiology of glomerulonephritis (GN). An allelic variant of *ITGAM* is a risk factor for systemic lupus erythematosus, including renal manifestations.^15^ Experiments in mice with homozygous deficiency of CR3 (*Itgam*^*–/–*^) have shown a deleterious role for CR3 in acute glomerular injury such as the heterologous nephrotoxic nephritis (NTN) model.^16^, ^17^ In contrast, a protective role for CR3 was demonstrated in experimentally triggered immune complex GN.^18^, ^19^ These differences have been attributed to “context-dependent” immune effects of CR3.^19^

The contribution of CR3 to the spontaneous renal pathology associated with FH deficiency is unknown. We investigated the effect of CR3 on the renal phenotype of *Cfh*^*–/–*^ mice by intercrossing these animals with the *Itgam*^*–/–*^ strain. We observed that CR3 deficiency exacerbated spontaneous renal disease associated with complete FH deficiency and that this was dependent on the expression of CR3 on bone marrow (BM)–derived cells. We also observed that the *Itgam*^*–/–*^ strain were more susceptible to renal damage during accelerated NTN (ANTN). The *in vitro* stimulation of CR3 with iC3b-coated targets limited the proinflammatory cytokine profile triggered by lipopolysaccharide (LPS) in murine and human macrophages. In contrast, cytokine secretion was enhanced in neutrophils, indicating that the modulating effects of CR3 differ between monocytes/macrophages and neutrophils. Together our data indicate that strategies to promote the interaction of CR3 with iC3b and other potential ligands in the kidney may be a suitable therapeutic goal in C3G.

## Results

### CR3 deficiency exacerbates spontaneous C3G in *Cfh*^*–/–*^ mice

**Figure 1:**
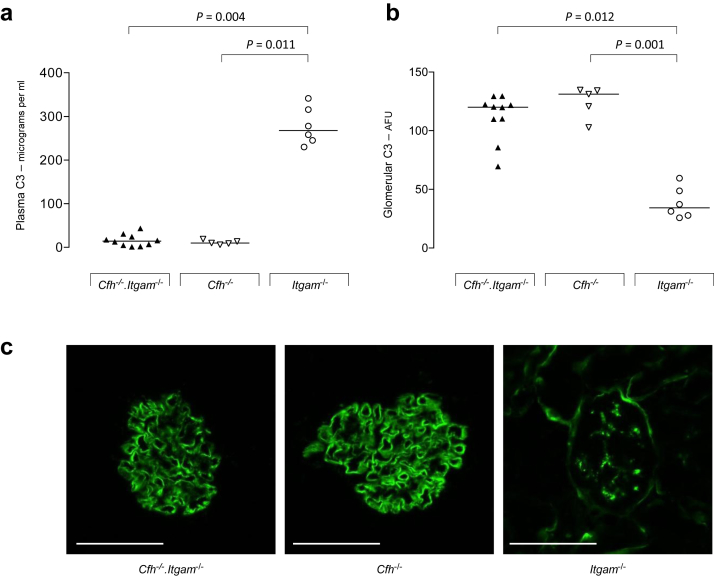
**Plasma and glomerular C3 in mice with combined deficiency of factor h (FH) and complement receptor 3.** (**a,b**) Plasma C3 levels (**a**) and glomerular C3 staining intensity (**b**) in 8-month-old *Cfh*^*–/–*^*.Itgam*^*–/–*^, *Cfh*^*–/–*^, and *Itgam*^*–/–*^ mice housed in specific-pathogen free conditions. Horizontal bars denote median values. AFU, arbitrary fluorescent units. (**c**) Representative images of glomerular C3 immunostaining in the 3 genotypes. Bar = 80 μm. Each symbol represents a mouse. *P* values derived from Dunn’s multiple comparison test.

The role of CR3 in experimental spontaneous C3G was assessed by generating mice with combined deficiency of FH and CR3 (*Cfh*^*–/–*^*.Itgam*^*–/–*^). Cohorts of female *Cfh*^*–/–*^*.Itgam*^*–/–*^ (*n* = 10) and *Cfh*^*–/–*^ (*n* = 5) mice were monitored in specific pathogen–free (SPF) conditions for 8 months. While plasma and glomerular C3 remained normal in *Itgam*^*–/–*^ mice, *Cfh*^*–/–*^*.Itgam*^*–/–*^ animals developed low plasma C3 and abnormal GBM C3 accumulation comparable to that seen in *Cfh*^*–/–*^ animals (Figure 1). However, *Cfh*^*–/–*^*.Itgam*^*–/–*^ animals developed greater renal inflammation at 8 months (Table 1). At this time point albuminuria was significantly increased in *Cfh*^*–/–*^*.Itgam*^*–/–*^ compared to *Cfh*^*–/–*^ mice (median 173.2 μg/16 h, range 72.5–987.2, vs. 74.8 μg/16 h, range 33.8–141.2, *P =* 0.028, Mann-Whitney test) together with greater glomerular cell counts (Table 1). A single *Cfh*^*–/–*^*.Itgam*^*–/–*^ mouse developed hematuria and distress necessitating killing at 6 months. In this mouse only, diffuse crescentic GN was evident on light microscopy. In a second cohort of female *Cfh*^*–/–*^*.Itgam*^*–/–*^ (*n* = 12) and *Cfh*^*–/–*^ (*n* = 12) mice housed in non-SPF conditions over an 8-month period, we noted significantly reduced survival in *Cfh*^*–/–*^*.Itgam*^*–/–*^ animals (Table 1). Renal histology was not available from the 6 *Cfh*^*–/–*^*.Itgam*^*–/–*^ animals that died prior to the 8-month time point. Of the 6 *Cfh*^*–/–*^*.Itgam*^*–/–*^ animals culled at the 8-month time point, hematuria and albuminuria were present in 4. Glomerular cellularity was similar between the *Cfh*^*–/–*^ and *Cfh*^*–/–*^*.Itgam*^*–/–*^ groups, but the number of glomerular macrophages was significantly increased in the *Cfh*^*–/–*^*.Itgam*^*–/–*^ mice (Table 1). These data indicated that CR3 deficiency increased mortality and spontaneous glomerular inflammation associated with FH deficiency and that the size of this effect was influenced by the environmental conditions. We next determined whether CR3 on BM-derived cells was responsible for this effect.

### Lack of CR3 expression on BM-derived cells exacerbates spontaneous C3G in *Cfh*^*–/–*^ mice

**Figure 2:**
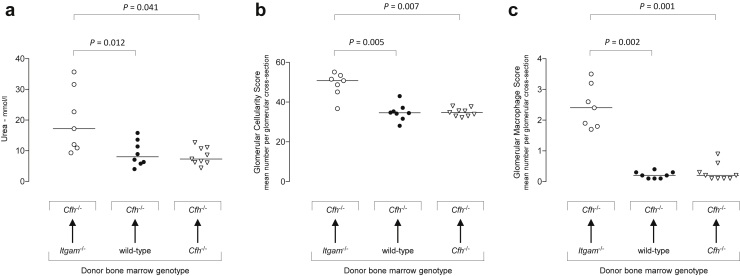
**Bone marrow transplantation in factor H (FH)–deficient mice.** Two- to three-month-old *Cfh*^*–/–*^ mice were reconstituted with bone marrow (BM)–derived cells isolated from *Itgam*^*–/–*^, wild-type, or *Cfh*^*–/–*^ mice and the spontaneous renal phenotype assessed at 8 months of age. Plasma urea (**a**), glomerular cellularity score (**b**), and glomerular macrophage score (**c**) were significantly higher in *Cfh*^*–/–*^ mice reconstituted with *Itgam*^*–/–*^ BM-derived cells. Horizontal bars denote median values. Each symbol represents a mouse. *P* values derived from Dunn’s multiple comparison test.

Three groups of irradiated *Cfh*^*–/–*^ mice were reconstituted with *Itgam*^*–/–*^ (*n* = 8), wild-type (*n* = 7), or *Cfh*^*–/–*^ (*n* = 9) BM-derived cells. At 8 months, *Cfh*^*–/–*^ recipients of *Itgam*^*–/–*^ BM-derived cells showed significantly increased plasma urea, glomerular cellularity, and glomerular macrophage counts (Figure 2). Notably, renal parameters between recipients of wild-type and *Cfh*^*–/–*^ BM-derived cells did not differ. These data suggested that the deleterious effect of CR3 deficiency on the renal disease associated with FH deficiency was mediated by the lack of CR3 expression on BM-derived rather than intrinsic renal cells. We next explored the effects of CR3 deficiency on experimental renal disease independent of FH deficiency.

### *Itgam*^*–/–*^ mice are more susceptible to ANTN

**Figure 3:**
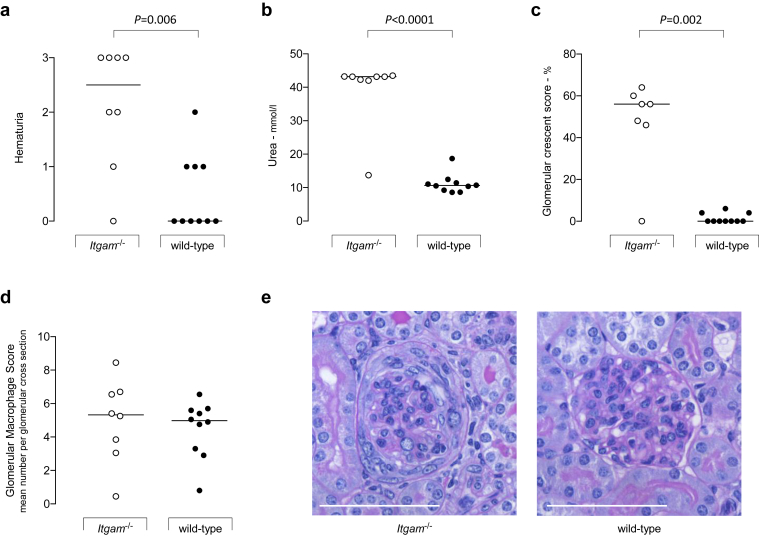
**Accelerated serum nephrotoxic nephritis in complement receptor 3–deficient mice.** (**a–c**) Hematuria (**a**), plasma urea (**b**), and glomerular crescent score (**c**) were significantly increased at day 10 in *Itgam*^*–/–*^ mice. One point is missing from the *Itgam*^*–/–*^ glomerular crescent data set due to loss of a sample. (**d**) Glomerular macrophage numbers, determined by CD68 reactivity, did not differ between *Itgam*^*–/–*^ and wild-type mice. Horizontal bars denote median values. Each symbol represents a mouse. *P* values derived from Mann-Whitney test. (**e**) Representative light microscopic images from *Itgam*^*–/–*^ and wild-type mice of glomeruli stained using periodic acid–Schiff reagent. A circumferential crescent is evident in the *Itgam*^*–/–*^ section, while the wild-type glomerulus appears normal. Bar = 80 μm.

*Itgam*^*–/–*^ mice showed no evidence of an abnormal spontaneous renal phenotype at 8 months in either SPF (*n* = 6F) or non-SPF (*n* = 10F) conditions. Specifically, hematuria did not develop in these animals, and glomerular histology at 8 months by light microscopy was normal (data not shown). We then speculated that CR3 deficiency could influence the response of the kidney during experimentally triggered renal inflammation. To investigate this we performed ANTN in *Itgam*^*–/–*^ (*n* = 8) and wild-type (*n* = 10) mice. Two days after injection of nephrotoxic serum, hematuria was detectable in 7 of the 8 *Itgam*^*–/–*^ mice but absent in all wild-type animals. Ten days after administration of nephrotoxic serum, when all animals were culled, the *Itgam*^*–/–*^ mice had significantly greater hematuria, plasma urea levels, and glomerular crescents (Figure 3). Glomerular macrophage counts were no different between groups. Glomerular deposition of sheep and mouse IgG and antigen-specific plasma titers of mouse IgG did not differ between the groups (data not shown). These data indicated that CR3 deficiency exacerbated renal injury during ANTN.

### iC3b ligation of CR3 on myeloid cells results in cell-specific cytokine release

**Figure 4:**
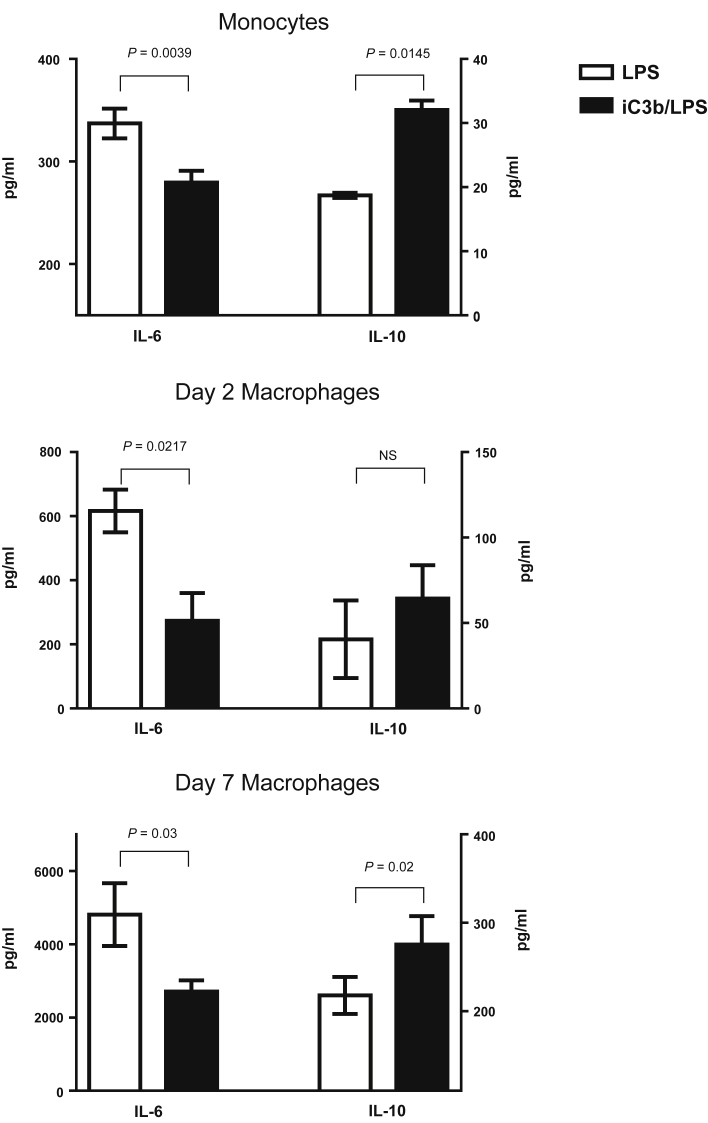
**Modulation of TLR4-induced cytokine release by iC3b-guinea pig red blood cells (gRBCs).** Murine monocytes (*n* = 3) (top), day 2 monocyte-derived macrophages (*n* = 3) (middle), and day 7 bone marrow–derived macrophages (*n* = 6) (bottom) were fed with iC3b-gRBCs (at a 10:1 ratio, gRBCs/cells) 1 hour prior to lipopolysaccharide (LPS) stimulation (10 ng/ml) for 24 hours. The amounts of interleukin-6 (IL-6) and IL-10 in the samples with and without CR3 pre-engagement are shown with the *P* values indicated. Data are expressed as mean ± SEM, paired *t*-test. The data are representative of 3 independent experiments.

**Figure 5:**
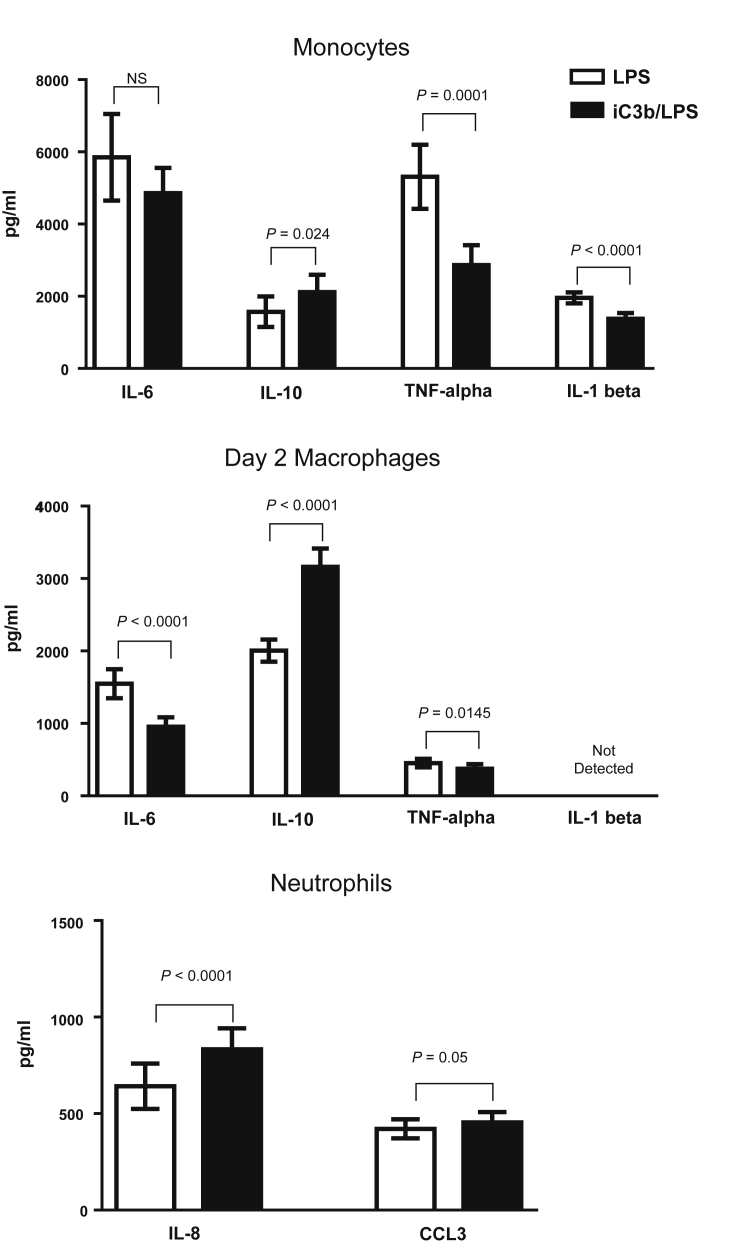
**Cytokine response of human cells to preincubation with iC3b-coated beads followed by lipopolysaccharide (LPS) stimulation.** Monocytes (*n* = 20) (top), day 2 monocyte-derived macrophages (*n* = 29) (middle), and neutrophils (*n* = 26) (bottom) were preincubated with iC3b-coated beads for 1 hour prior to LPS stimulation (10 ng/ml). Cytokines were quantified using a bead multiplex assay, and the levels with and without CR3 pre-engagement with iC3b are shown. Data are expressed as mean ± SEM, paired *t*-test. *P* values are indicated. IL, interleukin; TNF, tumor necrosis factor.

Since iC3b is abundantly present within the glomerulus in experimental C3G and complement is activated during ANTN, we hypothesized that the interaction between iC3b and CR3 on myeloid cells within the kidney resulted in an anti-inflammatory response that in turn reduced the severity of the renal injury. To investigate this we assessed the effect of iC3b-coated guinea pig red blood cells (gRBCs) on LPS-induced cytokine secretion by murine myeloid cells (both monocytes and macrophages at day 2 and day 7 of differentiation *in vitro*). Preincubation with iC3b-gRBCs resulted in reduced secretion of interleukin-6 (IL-6), but enhanced secretion of IL-10 (Figure 4). These cytokine changes were CR3-dependent since they were not observed when we used macrophages from *Itgam*^*–/–*^ mice (Supplementary Figure S1A online). We then examined human cells preincubated with iC3b-coated beads (Figure 5). We initially noticed that human monocyte-derived macrophages display a progressive loss of CD11b expression during differentiation *in vitro*, most likely due to adherence to plastic. By day 7, the expression of CD11b, including its active form assessed with the CBRM1/5 antibody, was barely detectable (Supplementary Figure S1B). Therefore, we chose to study monocytes and monocyte-derived macrophages at day 2 only. In both cell types iC3b pre-ligation downmodulated the proinflammatory cytokine profile triggered by LPS and promoted an anti-inflammatory response (Figure 5). In contrast, the cytokine effect of iC3b-coated beads on TLR4-stimulated neutrophils was proinflammatory with significantly increased production of IL-8 and CCL3 (MIP-1α). These cytokine effects were observed regardless of whether the cells were exposed to iC3b-coated beads before, simultaneously, or 1 hour after LPS challenge (data not shown). We also analyzed cytokine production after overnight stimulation with iC3b-coated particles alone or medium alone. Neither of these conditions induced detectable cytokines, demonstrating no endotoxin contamination (data not shown). Taken together these data demonstrate that the cytokine modulating effects mediated by CR3 through the binding of iC3b are distinct and cell type–specific.

## Discussion

Progressive renal disease in *Cfh*^*–/–*^ mice is associated with accumulation of C3 activation fragments including iC3b along the GBM. Here we show increased severity of the spontaneous glomerular phenotype in 8-month-old *Cfh*^*–/–*^ mice with coexisting deficiency of CR3, the leukocyte receptor for iC3b. Disease exacerbation was more marked in non-SPF compared to SPF housing conditions. This protective effect was dependent on CR3 expression on BM-derived cells. Glomerular macrophages were increased in *Cfh*^*–/–*^ animals reconstituted with *Itgam*^*–/–*^ BM-derived cells, suggesting that these macrophages were contributing to the protective effect. In order to determine whether CR3-dependent glomerular protection also occurred outside the setting of FH deficiency, we investigated the response of *Itgam*^*–/–*^ mice to ANTN. Early hematuria developed exclusively in *Itgam*^*–/–*^ mice, persisting until day 10 when these animals had evidence of severe renal injury with crescentic GN. These observations indicated that CR3 mediated a protective effect during subacute renal inflammation, that is, at a time when macrophages are present within the glomeruli.

CR3-mediated signaling is usually viewed as proinflammatory, and prevention of CR3–iC3b interaction by antibody or genetic deletion has been shown to decrease the severity of inflammatory responses in several animal models including heterologous NTN.^16^, ^17^ Hence the findings that CR3 provided a beneficial effect in our spontaneous model of C3G in *Cfh*^*–/–*^ mice were unexpected, and suggest that strategies to block CR3-dependent inflammation may not be beneficial as previously thought. An explanation for the contradictory role of CR3 in various disease models^10^, ^20^, ^21^, ^22^ may lie in the predominant role played by the different innate or adaptive immune cells in these experimental conditions and the fact that CR3 operates in a cell type–specific manner. Similarly, previous data in C3-deficient mice demonstrated that C3 contributed to renal injury in the acute phase of heterologous NTN.^23^ However, C3-deficient animals developed greater renal injury during the autologous phase of heterologous NTN. In the same study C3 deficiency was also associated with exacerbation of renal injury in ANTN. We speculated that in our model of spontaneous renal pathology associated with FH deficiency the renal protective effect was mediated by an interaction between iC3b and CR3 on BM-derived macrophages. We therefore examined the CR3-dependent cytokine responses of myeloid cells to iC3b-coated targets. We studied the macrophage cytokine response to TLR4 ligation, which plays a deleterious role in several models of GN, including ANTN.^24^ Here, we found that the interaction of iC3b with CR3 on both murine and human monocytes and macrophages is associated with an enhanced anti-inflammatory cytokine profile (reduced IL-6 and increased IL-10 secretion) following LPS stimulation. These data are consistent with earlier studies indicating that engagement of CR3 inhibited secretion of proinflammatory cytokines by TLR4-stimulated monocytes and macrophages.^10^, ^25^, ^26^, ^27^ Pre-ligation with a synthetic CD11b agonist has also been shown to inhibit human macrophage secretion of tumor necrosis factor-α in response to synthetic TLR7/8 ligands.^28^ Our data showing an anti-inflammatory effect due to iC3b ligation of CR3 on macrophages are also consistent with earlier studies showing downregulation of proinflammatory cytokines during phagocytosis of complement-opsonized apoptotic cells.^29^, ^30^ In addition, our data support the notion that the cytokine modulating effects of the iC3b–CR3 interaction are cell type–specific, since the effect on human neutrophils was proinflammatory. This opposite response mediated by CR3 to LPS stimulation may explain the conflicting results in the literature, including the deleterious role of CR3 in heterologous NTN, since this model mainly depends on the initial glomerular neutrophil influx.^16^, ^17^

Additional immune mechanisms may have contributed to our finding of CR3-mediated protection in the ANTN model. Firstly, ANTN has been extensively characterized in C57BL/6 mice as involving a T helper 1–predominant, delayed-type hypersensitivity–like nephritogenic immune response.^31^ T helper 17–directed cellular immune responses have also been implicated in several murine models of crescentic nephritis, including ANTN.^32^ Both enhanced delayed-type hypersensitivity and T helper 17 immune differentiation have previously been demonstrated in *Itgam*^*–/–*^ mice using a model of low-dose oral antigen (ovalbumin) exposure followed by high-dose immunization with antigen and complete Freund’s adjuvant (CFA).^21^ Secondly, CR3 may negatively regulate CFA-induced proinflammatory signaling, with renal injury in the ANTN model being dependent on the sensitization phase induced by IgG mixed with CFA. CFA is a recognized TLR agonist,^33^, ^34^ and increased renal injury due to TLR4 ligation specifically during sensitization has been demonstrated in ANTN studies.^24^ A protective role for CR3 was also reported in a passive transfer model of GN in lupus-prone (New Zealand black × New Zealand white)F1 with selective neutrophil transgenic expression of human FcγRIIA or FcγRIIA and FcγRIIIB.^19^ Again this model relied on a CFA-based protocol. In addition, CFA can induce a CD11b-positive splenic cell population with immunosuppressive effects on T cell–mediated immunity,^11^, ^35^ and this could also play an important role. Notably, these immune mechanisms do not appear to be implicated in the spontaneous GN occurring in systemic lupus erythematosus–prone MRL/MpJ-FAS^lpr^ mice, in which CR3 deficiency made no difference to the severity of the renal pathology.^36^

A recent study suggested that CR3 is a negative regulator of B-cell receptor signaling, based on experiments in anti-snRNP Ig transgenic mice.^37^ In our study we found that CR3 deficiency did not influence the antigen-specific IgG response following administration of nephrotoxic serum in sensitized (ANTN) mice, making an impaired antibody response an unlikely explanation for the protective effect mediated by CR3. Another study utilized the chronic serum sickness model of immune complex GN, in which mice were injected with horse spleen apoferritin.^18^ In that study, increased severity of proliferative GN in *Cfh*^*–/–*^ mice receiving an *Itgam*^*–/–*^ BM transplant, compared to *Cfh*^*–/–*^ recipients of either a wild-type or *Cfh*^*–/–*^ BM, correlated with an increased anti-apoferritin IgG immune response. However, chronic serum sickness without BM transplantation did not produce proliferative GN in *Itgam*^*–/–*^ mice (notwithstanding significantly increased albuminuria compared to wild-type mice), with no differences in the anti-apoferritin IgG immune response between the *Itgam*^*–/–*^ and wild-type groups. Notably, both previous studies^18^, ^37^ utilized repeated antigen dose administration (without adjuvant) over a long period, which is a different approach for eliciting IgG immune responses from the one used in our study.

We have shown that CR3 mediates renal protection *in vivo* during both spontaneous C3G and ANTN. In our model of experimental C3G, the interaction between the abundant iC3b along the GBM with CR3 expressed on macrophages appears to result in the downregulation of the proinflammatory cytokine response by macrophages. Mechanisms of renal injury in C3G include C3- and C5-dependent effector functions. Our data indicate that the iC3b–CR3 interaction does not contribute to renal injury. In contrast, this interaction is protective. Potentiating the macrophage CR3–iC3b interaction could therefore be beneficial in the treatment of C3G. Preclinical evaluation of small-molecule CR3 agonists has produced promising results in a number of animal models of glomerular disease including heterologous NTN.^38^ In summary, our data contribute to our mechanistic understanding of C3G and demonstrate that the anti-inflammatory consequences of the macrophage CR3–iC3b interaction result in reduced renal injury.

## Materials and Methods

### Mice

C57BL/6 wild-type mice were purchased from Harlan Ltd. (Bicester, UK) and CR3-deficient (*Itgam*^*–/–*^*)* mice^39^ from Jackson Laboratory (Bar Harbor, ME). FH-deficient (*Cfh*^*–/–*^) mice^3^ and *Itgam*^*–/–*^ were backcrossed onto a C57BL/6 genetic background for 10 generations. *Cfh*^*–/–*^*.Itgam*^*–/–*^ mice were generated by intercrossing the *Cfh*^*–/–*^ and *Itgam*^*–/–*^ strains. All animal procedures were performed in accordance with institutional guidelines and under license by the UK government.

### Accelerated NTN and BM transplantation

Accelerated NTN (ANTN) was induced by the i.v. injection of 200 μl of sheep nephrotoxic serum (a sheep Ig fraction containing anti-mouse GBM antibodies) into mice that had been sensitized with an intraperitoneal injection of 200 μg of sheep IgG (Sigma-Aldrich, Dorset, UK) in CFA (Sigma-Aldrich) as previously described.^3^ BM transplantation was performed as previously described.^40^ Briefly, mice were irradiated at 8 Gy using a ^137^Cs γ-ray source and reconstituted with 10^7^ BM-derived cells from donor strains. Mice were housed in SPF conditions and individually ventilated cages.

### Assessment of renal function, plasma C3, and immune response to sheep IgG

Dipstick hematuria was detected using Hema Combistix urine reagent strips (Siemens, Frimley, UK). Albuminuria was measured using radial immunodiffusion with a rabbit anti-mouse albumin antibody (product no. 0220-1829, AbD Serotec, Oxford, UK) and purified mouse albumin standard (Sigma-Aldrich) as previously described.^41^ Mouse blood samples were collected directly into ethylenediamine tetraacetic acid–containing Eppendorf tubes and kept on ice until centrifugation and stored as single-use aliquots at –80 °C. Plasma urea was measured using an ultraviolet method according to the manufacturer’s instructions (R-Biopharm, Glasgow, UK). Measurement of plasma C3 was performed by enzyme-linked immunosorbent assay (ELISA) as previously described^41^ using unconjugated polyclonal goat anti-mouse C3 capture antibody (product no. 0855463, MP Biomedicals, Cambridge, UK) and horseradish peroxidase–conjugated polyclonal goat anti-mouse C3 detecting antibody (product no. 0855557, MP Biomedicals). A standard curve was generated using acute-phase serum containing a known quantity of C3 (Calbiochem, Hertfordshire, UK). The mouse IgG anti-sheep IgG response was assessed by ELISA. Microtiter plates were coated with 3.5 μg/ml sheep IgG (Sigma-Aldrich), plasma samples added at doubling dilutions, and bound mouse IgG detected using a polyclonal sheep horseradish peroxidase–conjugated anti-mouse IgG antibody (product no. 515-035-062, Jackson ImmunoResearch Laboratories, West Grove, PA) and TMB substrate (BD Biosciences, Franklin Lakes, NJ).

### Histologic studies

Mouse kidneys were fixed in Bouin’s solution (Sigma-Aldrich), embedded in paraffin, and sections stained with periodic acid–Schiff reagent. Ten to twenty glomeruli per section were scored for total cells, crescents, and macrophages using light microscopy. Macrophages were identified by anti-CD68 antibody reactivity in either (i) acetone-dried frozen sections using Alexa Fluor 488–conjugated monoclonal rat anti-mouse CD68 (product no. 137012, BioLegend, San Diego, CA) to directly identify macrophages and biotinylated goat anti-peanut agglutinin polyclonal antibody (product no. BA-0074, Vector Laboratories, Burlingame, CA) with Alexa Fluor 555–conjugated streptavidin (product no. S-21381, Invitrogen, Carlsbad, CA) to identify non-glomerular structures; or (ii) frozen sections from renal tissue fixed in periodate lysine paraformaldehyde, washed overnight in 7% sucrose in phosphate-buffered saline (PBS) and frozen in isopentane precooled with liquid nitrogen using FA11 (monoclonal rat anti-mouse CD68, Serotec, Oxford, UK), polyclonal mouse anti-rat IgG, and rat peroxidase–anti-peroxidase applied sequentially (both from Jackson ImmunoResearch Laboratories).^42^ The slides were developed in diaminobenzidine and counterstained with hematoxylin (Sigma-Aldrich). Quantitative renal immunostaining for mouse C3, mouse IgG, and sheep IgG was performed on acetone-dried frozen renal sections using fluorescein isothiocyanate (FITC)–conjugated polyclonal goat anti-mouse C3 (product no. 0855500, MP Biomedicals, Solon, OH), FITC-conjugated polyclonal goat anti-mouse IgG Fc (product no. F5387, Sigma-Aldrich, St. Louis, MO), and FITC-conjugated monoclonal mouse anti-goat/sheep IgG (product no. F4891, Sigma-Aldrich, St. Louis, MO) antibodies as previously described.^43^

### Human and mouse cell isolation

Peripheral blood samples were obtained by venipuncture of healthy adult volunteers after informed written consent was obtained in accordance with the Declaration of Helsinki. Samples were collected as a subcollection registered with the Imperial College Healthcare Tissue Bank (NRES approval 12/WA/0196). Human neutrophils were isolated by dextran sedimentation and discontinuous plasma–OptiPrep gradients followed by negative selection using a custom antibody cocktail as previously described.^11^, ^27^ Monocytes were obtained by density gradient separation coupled with a negative selection kit for human monocytes as recommended by the manufacturer (Miltenyi Biotec GmbH, Bergisch Gladbach, Germany). Monocyte-derived macrophages were generated by culturing the cells for 2 days in RPMI-1640 medium supplemented with 10% fetal calf serum, 2 mM l-glutamine, 1% penicillin/streptomycin (Thermo Fisher Scientific, Waltham, MA), and 20 ng/ml recombinant human macrophage colony-stimulating factor (PeproTech, Rocky Hill, NJ) as described previously.^44^ Murine monocytes were purified from peripheral mouse blood with EasySep Mouse Monocyte Enrichment Kit (Stemcell Technologies, Vancouver, BC, Canada). Macrophages were generated by culturing murine monocytes with 20 ng/ml recombinant human macrophage colony-stimulating factor (PeproTech) for 2 or 7 days.

### Flow cytometry

Murine and human leukocytes were stained using standard protocols in the presence of a saturating concentration of 2.4G2 monoclonal antibody (anti-CD16/32). The following antibodies were used: phycoerythrin-conjugated anti-mouse/human CD11b (M1-70, product no. 12-0112, eBioscience, San Diego, CA) and phycoerythrin-conjugated anti-human CD11b (active epitope, CBRM1/5, product no. 12-0113, eBioscience). Data were acquired using a FACSCalibur (Becton Dickinson, Franklin Lakes, NJ) and analyzed using FlowJo software, version 7.6 (Tree Star, Ashland, OR).

### Cytokine assays

The cytokine assays were performed using human and mouse cells and 2 types of iC3b-coated particles: guinea pig red blood cells (gRBCs) for murine cells and Fluoresbrite carboxylate YG 1.5-μm microspheres (Polysciences Inc., Warrington, PA) for human cells. gRBCs (TCS Biosciences, Buckingham, UK) were opsonized with mouse C5-deficient serum at 37 °C for 30 minutes, resuspended to 1% vol/vol in culture medium, and added to mouse cells at a 10:1 ratio (gRBCs/cells). Fluoresbrite carboxylate microspheres were resuspended (1/200) in Krebs-Ringer PBS-Glucose buffer with human iC3b (20 μg/ml, Complement Technology Inc., Tyler, TX) and incubated at 37 °C for 30 minutes. Human iC3b–coated beads were then washed with PBS plus 1% bovine serum albumin plus ethylenediamine tetraacetic acid and resuspended in culture medium (1/400). For both iC3b-coated targets the level of iC3b opsonization was checked by flow cytometry using a biotinylated polyclonal antibody that recognizes both human and mouse C3 fragments (clone: 6C9, product no. CL7631B, Cedarlane Laboratories, Burlington, ON, Canada) followed by streptavidin-phycoerythrin (BD Biosciences Pharmingen). In each assay, neutrophils (2 × 10^5^ cells per well), monocytes (2 × 10^5^ cells per well), or macrophages (1 × 10^5^ cells per well) were preincubated for 1 hour at 37 °C with iC3b-coated targets and then stimulated with LPS 10 ng/ml (TLR grade R515, Enzo Life Sciences, Lausen, Switzerland). Cells incubated with iC3b-coated particles or medium alone were used as controls. Supernatants were collected after 24 hours and frozen until analysis. Human cytokine levels (IL-1β, IL-6, IL-10, tumor necrosis factor-α, IL-8, and CCL3) were measured with a bead multiplex assay (eBioscience) according to the manufacturer’s instructions and murine cytokine levels (IL-6, IL-10) with ELISA kits (R&D Systems, Minneapolis, MN).

### Statistical analysis

Statistical data were analyzed using Prism 6.00 for Windows (GraphPad Software, La Jolla, CA). *In vivo* data were analyzed using Mann-Whitney test for 2-group comparison, Dunn’s multiple comparison test for 3-group comparison, and log-rank test for survival analysis. *In vitro* data were analyzed using 2-tailed Student’s *t*-test for paired samples.

## Disclosure

MCP has received fees from Alexion Pharmaceuticals for invited lectures and for preclinical testing of complement therapeutics. All the other authors declared no competing interests.

## Figures and Tables

**Table 1:** Spontaneous renal disease in FH-deficient mice with or without CR3

	Specific pathogen–free conditions		Non–specific pathogen–free conditions	
Genotype	*Cfh*^*–/–*^*.Itgam*^*–/–*^	*Cfh*^*–/–*^	*Cfh*^*–/–*^*.Itgam*^*–/–*^	*Cfh*^*–/–*^
Mice, number/sex	10/F	5/F	12/F	12/F
Survival at 8 months, % (number)	90% (9)	100% (5)	50% (6)^c^	100% (12)
Plasma urea, median mmol/l (range)	13.3 (9.8–44.5)	11.5 (7.3–15.1)	13.5 (10.1–39.2)^d^	12.9 (9.7–25.7)
Glomerular cell count, median (range)	53.4 (40.8–75.9)^a^	36.6 (34.9–44.4)	44.2 (39.2–61.7)^d^	41.7 (35.9–56.1)
Glomerular macrophage count, median (range)	4.3 (0.9–9.9)	1.9 (1.2–3.1)	8.5 (5.1–14.5)^b^^,^^d^	3.7 (0.9–11.0)

F, female.

a *P* = 0.003. ^b^*P* = 0.0007, Mann-Whitney Test. ^c^*P* = 0.006, log-rank test. ^d^Data refer to the 6 mice that were culled at the 8-month time point.

## Supplementary Material

Supplementary Figure S1 CD11b is required for iC3b-mediated effect on LPS-induced cytokine production by myeloid cells. (**A**) Day 7 BM-derived macrophages from wild-type or *Itgam*^*–/–*^ mice were preincubated with iC3b-gRBCs for 1 hour prior to LPS stimulation (10 ng/ml). The amounts of IL-6 and IL-10 were measured by ELISA 24 hours later. The cytokine changes between the samples with and without CR3 pre-engagement with iC3b are shown with the *P* values indicated. Data represent mean ± SEM (*n* = 3). (**B**) Histograms showing cell surface expression of CD11b on human monocytes and monocyte-derived macrophages at day 2 and day 7 during *in vitro* culture. The expression was assessed by flow cytometry using 2 antibodies: ICRF44 and CBRM1/5 (active state). The data are representative of 2 independent experiments.
