# Supplementary material for: Complement receptor 3 mediates renal protection in experimental C3 glomerulopathy
Source: Kidney Int. 2016 Apr;89(4):823–32. doi: 10.1016/j.kint.2015.11.024 (PMC4869622; doi:10.1016/j.kint.2015.11.024)
Supplement: Supplementary Figure S1 — CD11b is required for iC3b-mediated effect on LPS-induced cytokine production by myeloid cells. (A) Day 7 BM-derived macrophages from wild-type or Itgam–/– mice were preincubated with iC3b-gRBCs for 1 hour prior to LPS stimulation (10 ng/ml). The amounts of IL-6 and IL-10 were measured by ELISA 24 hours later. The cytokine changes between the samples with and without CR3 pre-engagement with iC3b are shown with the P values indicated. Data represent mean ± SEM (n = 3). (B) Histograms showing cell surface expression of CD11b on human monocytes and monocyte-derived macrophages at day 2 and day 7 during in vitro culture. The expression was assessed by flow cytometry using 2 antibodies: ICRF44 and CBRM1/5 (active state). The data are representative of 2 independent experiments. [file mmc1.pdf]

Supplemental Data

Complement receptor 3 mediates renal protection in experimental C3 glomerulopathy

Thomas D. Barbour[1], Guang Sheng Ling[1], Marieta M. Ruseva[1], Liliane Fossati-Jimack[2], H. Terence Cook[1], Marina Botto[1], Matthew C. Pickering[1]

[1]Centre for Complement and Inflammation Research, Imperial College, London, United Kingdom; [2]Centre for Experimental Medicine and Rheumatology, Queen Mary University of London, London, United Kingdom

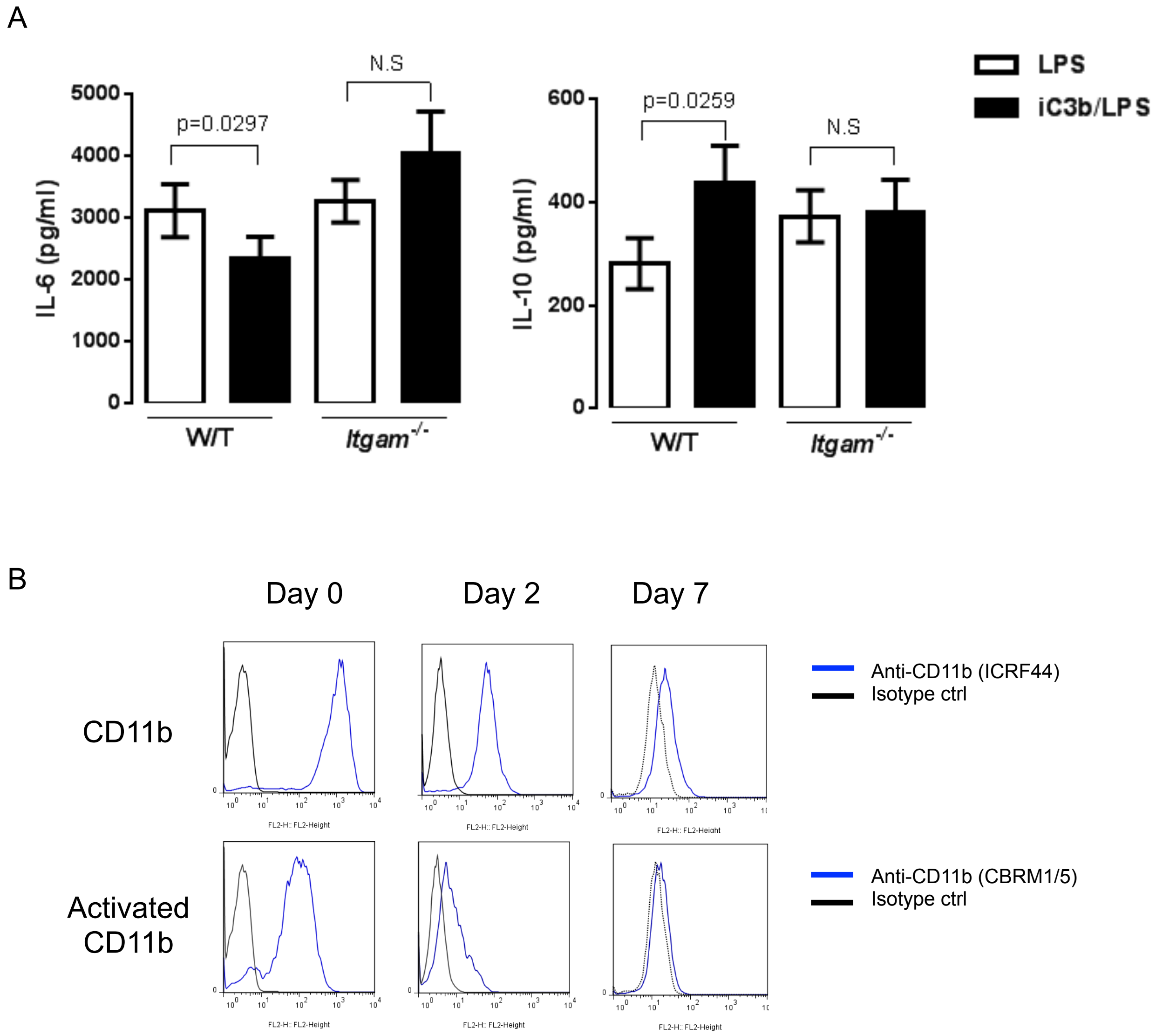

Supplemental Figure 1
